# *“You are a child and this is not your business”*: Decision-making on child marriage in Sindh, Pakistan

**DOI:** 10.1371/journal.pone.0266865

**Published:** 2023-09-29

**Authors:** Tasneem Kakal, Maryse Kok, Maryam Jawad

**Affiliations:** 1 KIT Royal Tropical Institute, Amsterdam, The Netherlands; 2 Independent consultant, Islamabad, Pakistan; University of Dhaka: Dhaka University, BANGLADESH

## Abstract

Young people in Pakistan face challenges such as child marriage, which have adverse consequences on their education, employment, health and overall well-being. We conducted interviews (26) and focus group discussions (12) with young people (15 to 24 years) and community stakeholders to understand how child marriage is perceived by them and to gain insight into the decision-making regarding marriage of youth in Sindh, Pakistan. Study findings show that many young people wish to marry later, but recognize that child marriage is used as a protective strategy to cope with poverty and prevent sexual activity prior to marriage. Young people are expected to obey elders and young women are relegated to domestic roles which limit their decision-making about marriage. Young people and parents who are educated seem to have more negotiating power in delaying marriage than those with little or no education. Our results suggest that interventions should focus on expanding education and livelihood opportunities for young women while changing social norms through parental engagement and youth empowerment.

## Introduction

Young people in Pakistan continue to experience child marriage with 18% of young women and 4.7% of young men between 20 and 24 years having married prior to age 18 [1]. Child marriage refers to any formal or informal union of a person under the age of 18 [2]. It limits educational opportunities of adolescents, particularly of girls, and adversely effects their health and empowerment due to the likelihood of adolescent pregnancies [3].

In Pakistan, some communities use marriage to protect adolescents against pre-marital relationships and sexual abuse; and as a way to maintain the family’s honour. Across South Asia, including in Pakistan, child marriage is also seen as a means to alleviate economic hardships of the family [4, 5]. Girls from households with poor education and low levels of income are more susceptible to child marriage, which is mostly arranged by parents [3, 5, 6]. In addition, the availability of suitable grooms (belonging to certain castes and religions, having economic capital and high educational qualifications) and funds for dowry are factors motivating child marriage [7]. Local traditional customs also drive child marriage [8].

As per the Child Marriage Restraint Act (CMRA), the national minimum age of marriage is 16 years for girls and 18 years for boys. In 2013, the minimum age of marriage for girls was increased from 16 to 18 years in Sindh province [9]. As per the Demographic Health Survey 2017–18, 29% of women (29–49 years) reported being married by the age of 18. The percentage of young women (15–19 years) in Sindh who reported to be married was 12.7% in 2012–2013 and 13.5% in 2017–2018 [10]. These rates could be attributed to the poor implementation of the CMRA and contextual factors such as economic constraints, and climate change [11]. In Sindh, 66–78% of marriages were (arranged) exchange marriages *(Watta satta)*, a traditional custom where a pair of siblings are exchanged in marriage between two households [12].

In the Pakistani context, young women are regarded as subordinate to men, have low decision-making power and have limited education and knowledge around sexual and reproductive health and rights (SRHR) [9, 13, 14]. For instance, in a study conducted by Nasrullah et al. (2014) in the urban slums of Lahore, 13 out of 19 of the women interviewed were not aware of the negative consequences of child marriage and were satisfied with their parents’ decisions to marry them before 18 years [8]. Hamid et al. (2009) interviewed 10 married girls (13–19 years) in a slum of Islamabad and found that they were unprepared for marriage as they had limited knowledge about SRHR [15]. The participants also reported that improved knowledge could help them to prepare for their future life [16]. In Sindh and Punjab, where only 34% of surveyed young women were consulted regarding their marriage, parents were found to be the major decision-makers regarding partner choice and the time at which girls could marry [17]. Understanding the dynamics of decision-making regarding marriage would help in developing context-specific and effective interventions. The Yes I Do programme (2016–2018) aimed at improving young people’s SRHR and reducing child marriage and teenage pregnancy in Sindh, Pakistan. A package of interventions, including a ‘whole school approach’ [18], community dialogues, and economic empowerment activities, was implemented in selected districts. In particular, the programme developed a *Kiran* network model comprising of peer educators who were trained on understanding SRHR and raising awareness about child marriage through door-to-door community campaigns. Drawing from data collected as part of the programme, this study explores how child marriage is perceived by community members, including young people (15–24 years) and provides insight into the decision-making dynamics regarding marriage of youth in Sindh, Pakistan.

## Methodology

This study was part of a broader qualitative (midline) study that was conducted to inform the Yes I Do programme in Pakistan. The programme was jointly implemented by Rutgers Pakistan, Plan Pakistan and Sindh Agricultural Forestry Workers & Coordinating Organization (SAFWCO), while KIT Royal Tropical Institute was the research partner. The programme was meant to run until 2020, but it ended in 2018 owing to changes in the government’s policy regarding international non-governmental organizations (NGOs) working in Pakistan. Data collection for the study was done in May, 2018, two years after programme implementation. Data were collected from two sub-divisions of Sanghar district (Sanghar and Jhol) and one sub-division of Umerkot district (Umerkot).

### The context

In a Muslim majority nation, both districts, especially Umerkot, have a relatively large population of Hindus. Sanghar and Umerkot districts are pre-dominantly rural. As per data between 2012 and 2014, both districts are relatively poor, particularly Umerkot, which has the highest intensity of poverty in Sindh [19]. Umerkot is adjacent to the Indian border and security concerns have worsened its socio-economic development [20]. Additionally, due to extreme climatic conditions (which resulted in a drought in Sindh since 2013), Sanghar and Umerkot were categorised as being in the ‘Emergency Phase’ in regards to acute food insecurity at the time of the study [21]. Government education data show that as of 2014–15, Sindh has primary schools and only a few higher secondary schools. Completion rates for primary school for boys is 52% in Sanghar and 57% in Umerkot, while for girls, it is 38% in Sanghar and 26% in Umerkot [22]. In 2016–17 in Sindh, among children between 5 and 9 years (primary-school age), 31% were out of school with the majority being girls [23]. Labour force participation, which is 30%, also favours men [24]. Since bonded labour practices are common in Sindh [25], 7,00,000 children are estimated to be in bonded labour [26]. Many of these labourers belong to lower-caste Dalit Hindu families [27]. In most cases, tenancy relations are inherited and all family members including women and children are expected to work in the fields [28]. In rural Sindh, most females (many whom are between 10 and 14 years of age) are engaged in unpaid agricultural work [29]. According to our prior research, 30% of young women and 25% of young men in Sanghar and 40% of young women and 13% of young men in Umerkot (all between 18 and 24 years) marry before the age of 18. Awareness about the CMRA is low and its implementation is poor [5]. Lastly, 11.2% of girls between 15 and 19 years of age experience early childbearing in rural Sindh [1].

### The study methods

We chose to use qualitative study methods to ensure an in-depth insight into the ‘why’ and ‘how’ of child marriage [30]. Data were collected through 12 focus group discussions (FGDs), 20 in-depth interviews (IDIs) and six semi-structured key informant interviews (KIIs) (Table 1). More FGDs, IDIs and KIIs were conducted in Sanghar because the Yes I Do programme had a greater focus on Sanghar as compared to Umerkot district.

**Table 1:** Detailed overview of study participants in FGDs, IDIs and KIIs.

Study methods	Participants		Number of FGDs/ interviews	Total number of study participants
**FGDs**	Young men	15–19 years, married	1	93
		15–19 years, unmarried	1	
		15–24 years, married	1	
		20–24 years, unmarried	1	
	Young women	15–19 years, married	2	
		15–19 years, unmarried	2	
		20–24 years, married	1	
		15–24 years, unmarried	1	
	Parents/ caregivers	Mothers	1	
		Fathers	1	
**IDIs**	Young men	15–19 years, unmarried	1	20
		20–24 years, married	1	
		20–24 years, unmarried	2	
	Young women	15–19 years, married	1	
		15–19 years, unmarried	1	
		20–24 years, married	1	
		20–24 years, unmarried	1	
	Parents/ caregivers	Mother	1	
		Father	1	
	Grandparents	Female	2	
	Health workers	Female	1	
		Male	1	
	Teachers	Female	1	
		Male	1	
	Religious leaders	Male	2	
	Young community-based	Female	1	
	organization representatives	Male	1	
**KIIs**	District level policy makers/ legislators	Male	3	6
	NGO staff	Male	3	
**Totals**			**38**	**119**

The FGDs and IDIs were conducted with young women and men (15–24 years) and parents or caregivers. Other IDIs were conducted with health workers, religious leaders, teachers and staff of community-based organizations (CBOs). KIIs were conducted with policy makers, legislators and NGO staff at district and national level. Each FGD had between 6 to 8 participants. Interviews and FGDs were conducted in private settings, such as participants’ homes or classrooms.

The research team consisted of the authors and four research assistants (two females and two males). The research assistants were graduate students in social science, were fluent in Urdu and Sindhi and had prior experience in conducting qualitative research. They were trained on the research objectives, sampling methods, research tools and ethics prior to data collection.

A topic guide for FGDs and IDIs and a guide for KIIs were developed by the research team, in collaboration with programme partners. The guides were translated into Urdu and Sindhi and were pre-tested. The topic guides focused on participants’ experiences, opinions and feelings about SRHR–including child marriage and teenage pregnancy, the related social norms, community and youth participation in decision-making, opportunities for schooling and economic empowerment, and SRHR-related policies and laws. While the topic guides for FGDs with youth, parents and caregivers focused more on group norms, the IDI topic guide dived into the participants’ personal experiences and opinions. The topic guide for KIIs dealt with child marriage policies, interventions and activities taking place at the community level.

Purposive sampling was adopted to select study participants based on age, gender and marital status. For the FGDs and IDIs, young people between the age of 15 and 24 years, who had been exposed to Yes I do programme activities were sampled. This included young people who were in school or out of school. In-school youth were chosen from 20 schools that were randomly selected from a longer list of schools where the Yes I Do programme was being implemented. The out-of-school youth and other study participants were selected from the community in which the schools were situated.

Interviews were audio-recorded with consent and research assistants took field notes. FGDs lasted about 1.5 hours and interviews lasted about an hour. One of the authors supervised and supported the team of research assistants to ensure the quality of the collected data. At the end of each day of data collection, the research team debriefed to assess the quality and progress of the data collection and discuss any challenges they encountered. The research team based in Pakistan was in regular virtual contact with the authors at KIT to discuss any challenges and opportunities encountered during data collection. All data were transcribed verbatim and translated to English. One researcher conducted quality checks on random transcripts against the audio files.

A thematic content analysis was conducted using Nvivo version 12. A generic coding framework was developed based on recent and relevant literature and findings from the baseline conducted in 2016 [5]. Emerging themes from the data were discussed among the research team and added to the coding framework. The coding process helped to identify patterns (similarities or differences) in the responses of the study participants from both districts. Narratives were written according to main themes and sub-themes.

A No Objection Certificate (NOC) was obtained from the Deputy Commissioner of Sanghar–Social Welfare Department for the research in Sanghar and a letter of approval from the Office of the Deputy Director Social Welfare Umerkot was obtained for Umerkot. As part of the consent process, the research team explained the objectives of the study, possible risks and benefits, and emphasised the participants’ right to withdraw their consent at any moment during the interview, without any penalties. Consent forms in Sindhi and Urdu were made available to participants. However, participants gave informed oral consent since they felt uncomfortable sharing their names or signing consent forms. In the case of minors, consent from their parent or caregiver was sought along with the minor’s assent. The larger Yes I Do study protocol which included the midline in Pakistan was approved by the Research Ethics Committee of the KIT Royal Tropical Institute (Proposal S69).

## Results

### Demographic characteristics of the sample

The gender of study participants is specified in Table 1. A mix of married and unmarried youth participated in the study. Some identified as Hindu and others as Muslim. They had varying education levels with some having no formal education. Most of the fathers who were interviewed in this study were relatively well-educated and many of them worked as teachers. Meanwhile, all the mothers that were interviewed had no formal education and primarily did care work in their home. The two grandmothers interviewed had no formal education and were 50 years old. Of the two religious leaders (one Muslim and one Hindu) interviewed, one had a graduate degree while the other had completed primary education. Both health workers interviewed were above 40 years of age and were educated (matric level and higher). Similarly, both teachers interviewed had a higher education degree. Two CBO representatives (aged 20 years and 28 years) were interviewed. All key informants interviewed were male, above 40 years of age and identified as Muslim.

### Perceptions of and motivating factors for child marriage

While many young people and some adult participants acknowledged the societal value of child marriage, they themselves did not see any benefit in the practice. In contrast, some young and adult participants believed that child marriage was justified due to a variety of factors—economic benefits for the natal household, conjugal benefits for the in-laws, the ‘protection’ it offered young women, and for young women to successfully conform to the societal expectations placed on them.

#### Children, particularly girls are considered a burden

The discourse that children were considered to be a (financial) burden was frequently invoked by young women. According to a young woman (FGD, 15–19 years), *“Parents consider them [daughters] as burdens so at an early age*, *she gets married*. *And we [daughters] do spare the parents from the responsibility [when we marry early]*.*”* The financial burden of raising a young woman who would eventually marry and move to the in-laws home was also mentioned by a teacher and some youth. A young man (IDI, 20–24 years) summarized the sentiment when he stated that parents would save on expenses if their daughter was married at a young age. Meanwhile, a mother (FGD) and a young woman (FGD, 15–19 years) spoke of the pressure on young men to fulfill expected responsibilities to financially provide for their families. A young woman (IDI, 15–19 years) referred to unmarried sons as (potential) ‘burdens’ on the family, while married sons could be independent.

#### Child marriage perceived as protecting girls against pre-marital sex and sexual harassment

Some community members perceived child marriage to be a viable strategy to hinder young women from consensual pre-marital sex and to protect against sexual harassment. For a young man (IDI, 20–24 years), marriage protected young women from ‘bad habits’. According to a young woman from a youth organization, community members believed that that marriage freed girls from ‘negativity’. A grandmother elaborated on this with an anecdote about a young woman with a boyfriend. The girl’s parents feared that she would elope with her boyfriend and got her married to another man to prevent her from eloping. The same grandmother also drew a clear distinction between the behaviour of educated and uneducated young women. She berated young women who had no education and remained unmarried by the age of 18 years. According to her, this increased the chances of girls starting to meet boys (dating). This was, apparently, not a problem with educated girls. A key informant from an NGO also pointed to child marriage as a strategy to protect girls against sexual harassment. These motivations for child marriage explained why young people married (or were married) early, even when they preferred to marry after 18 years of age.

#### Marriage, children and chores as a part of life

Some youth mentioned that society perceived child marriage as beneficial, because the young bride would take care of the husband, the in-laws and assist in the household chores. For a young woman (IDI, 15–19 years), marriage was a social arrangement that was part of life: girls would get married, go to their in-laws’ house to care for them and assist them with household chores. A young man (FGD, 20–24 years) said that as long as the couple was “settled” (had a stable income and clear living arrangements), there would not be negative consequences of child marriage. Another young man emphasized that a young couple could have children early, and their children would help to support them later in life. A young woman and two adults spoke of *Watta satta* marriages being common. Bringing a baby into the family and learning how to live responsibly in society were other perceived benefits of child marriage.

### Decision-making dynamics regarding marriage

In most cases, the mandate of deciding on children’s marriages rested with the parents. Young people, particularly women, did not have the option of making these decisions. The educational status of both the parents and the children influenced the likelihood of a child marriage in the household. The study also documented the community’s idea of consent. Some young people were able to resist their own marriage and intervene in other young people’s marriages. This was particularly true in cases where they were exposed to or trained through the Yes I Do programme.

#### Parents are the primary decision-makers

Almost all participants reported that parents were the primary decision-makers concerning young people’s marriage. Two key informants explained that fathers played the biggest role in making this decision, given the patriarchal societal setup. Other (male) relatives were mentioned as decision-makers by young men (FGD, 15–19 years) and by a grandmother (IDI). In a context where most marriages were registered solely at the religious office, a few youth–mainly young women, spoke of ‘*pandits*’ or religious priests falsifying ages of the couple and officiating child marriages.

Several young people, some of whom had participated in the Yes I Do programme, felt that it was important for young people to be able to make their own decisions. They reported that parents were increasingly seeking their children’s consent before arranging marriages. A young woman (FGD, 15–19 years) linked this change to an increased awareness about negative consequences of child marriage. Most youth, and all parents explained that parents would not forcefully marry their children off. However, a father in an FGD still estimated that only 10% of the parents asked for their children’s opinion. Several participants reiterated the influence of education and agreed that educated parents gave their children a higher degree of freedom and choice when it came to marriage-related decisions.

*“In some communities*, *children are not being consulted for marriage*, *parents make the decision and fix the date of marriage*. *Even before their marriage*, *they cannot see each other [referring to the couple]*, *but in educated communities*, *the situation is different*. *They ask the wish of the girl regarding marriage*, *and the right should be given to boys and girls to decide on their marriage*. *In our surroundings*, *50% of the people accept the right of children and 50% do not*. *People who have the awareness*, *they respect the rights of children*.*”* Young woman from a youth organization, 20 years, IDI

According to a father (FGD), the decision to marry should primarily be in the hands of the child, along with the opinion of the parents. It should be noted here that most of the fathers interviewed in the FGD were highly educated and were working as teachers. This could have influenced some of these opinions. Some of the fathers stated that there would be no negative consequences if a young man refused marriage. Another father recognized that giving young people space to choose their partners would mean that girls would not run away from home, and that they would confide in their parents. This was mentioned in a context where girls eloping with boys was stigmatized and brought shame to the family. The FGD conducted with mothers who had more diverse backgrounds and varying education levels compared to the fathers added some nuance to the findings. According to them, although mothers were consulted and young people could inform their parents if they disagreed, the father had the final say in the matter. As per a health worker, even if parents were educated, this did not did not automatically guarantee children’s consent being considered.

*“I have seen some people who get the consent of children regarding their marriage*, *even when having a low [level of] education*. *But lots of educated people also ignore the desire of children*.*”* Female health worker, IDI

#### Young people have little to no space in making decisions about their marriage

Many young people clearly stated that they could not make decisions regarding marriage themselves and they had to seek permission from their fathers. A policy-maker highlighted the influence of gender norms and invoked the discourse of girls being a ‘burden’, when positing that girls were expected to be married in the future, and this idea influenced the way in which girls were raised.

*“They [parents] take them [daughters] as a burden*, *not as a responsibility*. *First they give education to their children*, *after that they get them married without even asking them*, *especially girls*.” Young man, FGD, 15–19 years

There were marked differences between the decision-making capacities and available freedom granted to young women and young men. A young woman (IDI, 20–24 years) remarked that if girls refused marriage, they would feel bad for their parents and a female teacher clearly indicated that girls could not refuse marriage:

“*Parents take the decision*, *if any boy denies the decision of his parents*, *he can be thrown out of the house*. *Parents call the boys bad-mannered*. *But girls cannot deny the decision of their parents*.*”* Mother (also a teacher), IDI

Most mothers (FGD) had mixed opinions on whether boys had more decision-making space compared to girls. One of the mothers opined that while some parents would ask the boys for their consent, this was not the case for girls.

Similar to educated parents, youth with education displayed a greater say in the decision-making process. According to a grandmother, a young woman with education would be respected by her community and her parents would not get her married before she turns 18 years. A father (FGD), remarked that an educated girl, when married would also have more bargaining power within the marriage. A few youth and adult participants also spoke of the potential income (which would be earned after education) and economic security as a clear benefit of educating girls and not getting them married. This was also mentioned in the context of vocational training or skill-building opportunities that could translate into jobs and in effect, prevent child marriage.

*“If a girl is a doctor*, *teacher*, *her parents will not arrange her marriage at an early age*, *because she is the source of income*, *she is earning and giving [her income] to her parents*, *then why would they arrange her marriage early*. *In villages*, *the situation is different*, *the acceptance of girls’ education is that girls study a little bit*, *and then they marry her off*.*”* NGO participant, KII

When participants discussed making decisions about marriage, the range of what constituted girls’ consent was considered particularly important. It seemed that young women’s participation in decision-making was limited to giving consent for marriage, and only in a few cases extended to choosing a partner. A few young woman (IDI, 15–19 years and FGD, 15–19 years), stressed that the marriage would not last without mutual consent and understanding and could lead to a divorce. One key informant questioned the ability of young people to give meaningful consent.

*“See if a child is 12 or 13 years old and he/she is getting married*, *it’s out of question to ask them about their will*. *So they even sometimes don’t know what marriage is*, *so they also don’t know what decision-making is*, *they don’t know what responsibilities they have to handle; it is just a change of home*, *one home to another home*.*”* NGO participant, KII

#### A few young people are refusing, resisting and intervening in cases of child marriages

The study found cases of child marriage where there was refusal or resistance. In some instances, it led to the marriage being stopped. Almost all participants explained that with increasing awareness, there were changes in the attitudes of parents and community members towards child marriage. As mentioned by a young woman from a youth organization, this increasing awareness and knowledge meant that people were able to resist child marriage. When discussing the role of youth clubs, a young woman (FGD, 15–19 years) testified to the influence of the Yes I Do programme by sharing how she had stopped her own marriage:

*“Yes they [youth clubs] prepared the youth a lot, now the youth is aware and they can raise their voice for their rights. I myself refused when my parents fixed my marriage when I was in class five. I told them that this is not my age of marriage. I am still a child and I don’t know what marriage means. My father wanted to marry me because he was not well and his financial condition was also not good.”*

Education, as mentioned earlier, also played an important role in resisting child marriage.

*“…educated girls and boys can also resist the decision and have the final say*.*”* Grandmother, IDI

A young man (IDI, 20–24 years) took a stand that child marriage could be stopped by informing parents that it is a crime, and if needed, the matter could be taken to court. He gave two examples in the Bhel and Mengwar communities, where a child marriage was stopped 10 days prior to the marriage ceremony. The strategy of going door to door and sensitizing parents was primarily done by *Kirans* (peer educators) from the Yes I Do programme. Many study participants specifically mentioned the *Kirans* as playing a crucial role in raising awareness. A key informant from the programme shared that they had 62 cases in which the marriage was delayed and none of the 800 *Kirans* were married prior to 18 years of age. A young woman (FGD, 15–19 years) who was also a *Kiran* described the process of intervening in case of a (planned) child marriage in the community. The *Kirans* would first approach the parents, and if they did not listen to them, they would approach the elders. Approaching the police was used as a method of last resort. Another young woman (IDI, 15–19 years) had stopped many child marriages by informing parents that it was a criminal offence and that both families were punishable by law. There were other efforts at the community level as well. According to a father (IDI), there was a committee set up by the village to stop early marriages. He explained that *“we have tried and stopped many marriages and are raising awareness [among] parents about issues relating to these marriages”*. Some youth and adult participants mentioned informing the police since child marriage is illegal. As recounted by a key informant (NGO), some villagers in Umerkot reported a case of child marriage to a female police officer who managed to stop the marriage.

A young woman highlighted that while young people–in particular boys–could refuse marriage, the reason for refusal had to seem ‘genuine’. If parents were not convinced by the refusal, they would wonder if the boy was engaged in a pre-marital relationship. Another young woman (FGD, 15–19 years) shared that parents would interrogate the boy if he refused marriage, as they considered him indebted to him for all that they had paid for his education. Other young people spoke of resisting child marriage but did not clarify the exact process further. Two young women (FGD, 15–19 years) referred to another girl in their FGD whose early marriage was stopped and reiterated that there was a change in the community.

According to a grandmother, young people could approach their relatives for help if they were being forced to marry by their parents. However, a young woman (IDI, 15–19 years) opined that community members would often remain silent when encountering cases of forced marriages. This was because parents were seen to have the ultimate right to decide on behalf of their children. Some young people also faced backlash when intervening to stop others’ child marriages. A young man relayed an incident where he asked his relatives not to marry their child at an early age, but he was reprimanded and the relatives reminded him that he was “a child and it was not his business”. A young man (FGD, 15–19 years) shared similar difficulties in speaking up against child marriage to elders who were uneducated and unaware of the law.

*“In my community a case of child marriage occurred*, *but what can they do in front of the elders*? *If we tell them*, *they say ‘what’s wrong with you*?*’ They are right*, *they don’t know about the laws because they are uneducated*.*”* Young man, FGD, 15–19 years

## Discussion

Study participants consistently referred to the influence of education on the likelihood of child marriage. The educational level of young people, particularly of young women and that of parents, plays a role in delaying child marriage. Given the limited presence of higher education institutions in both districts, and gender norms that frame girls as burdens and limit their mobility, it is not surprising that only one out of four young women in Sanghar and Umerkot had some form of education [5]. Being educated, particularly for young women, gives them leverage to be ‘valued’ members of the family as opposed to being a ‘burden’ and being married as a child in a context of dire economic insecurity. A study in neighbouring India found that limited education and poverty remain the most influential factors for child marriage, and educational and economic empowerment would be powerful strategies to counter this [31]. However, findings by Raj et al. (2014) demonstrate that while secondary education reduced the likelihood of girls marrying early, the effects were modest since many young women married as minors at later ages of adolescence [32]. Moreover, the effects of education are limited in contexts that have strongly ingrained unequal gender norms [33]. This is also relevant in the patriarchal context of Pakistan where the median age of marriage for women (25–49 years) is 20.4 years [1]. In India, a study found that a promising marriage proposal can overpower the potential future benefits of education [34]. In the context of Sanghar and Umerkot districts, with food insecurity, bonded child labour, poverty, weak education systems and limited employment opportunities, the approach to prevent child marriage must be multi-pronged [5]. There is a need to advocate for girls’ education and expand their livelihood options, while working on wider social norm change.

Our study found that the benefits of education also apply to parents, where parental education and aspirations are associated with delays in marriage, as also found in Senegal, Ethiopia and India [35–38]. However, other research has shown that while parents felt that young people had the right to make decisions, they doubted their capabilities to do so. Parents in Pakistan usually controlled major life decisions of young people, particularly of young women [39]. Other studies have also found that when parents ask for the consent of the girl before her marriage, it is often tokenistic [34, 40]. Our study also shows that among the few parents who asked their daughter for consent, this was often limited to whether she wanted to marry and rarely extended to whom she would want to marry. However, parents seeking their children’s consent on the timing of marriage might demonstrate gains in a context where most marriages are arranged by parents. Our study did not encounter any young woman who chose her partner. However, an increase in educational levels of young women has been associated with an increase in likelihood of young women choosing their partners in arranged marriages in India [41]. Crivello et al. (2018) found that girls often agreed to marriage in order to secure family support in the event of marital problems in the future [34]. In the Pakistani context, where the young people are expected to obey elders, engaging parents (through adult peer education and inter-generational dialogues) and promoting alternative parenting styles may offer gains in delaying marriage [42–44]. Our research on child marriage across seven countries had similar findings. We found that programmes focusing on community engagement need to continue engaging parents to bridge the inter-generational gap, particularly while discussing sensitive topics [45].

Moreover, to counter harmful gender norms that restrict young women to domestic roles and limit their educational and livelihood options, interventions focusing on social norms could offer opportunities for change [33]. The finding that child marriage functioned as a protective and preventive strategy against forced and premarital sexual activity also speaks of a taboo on (young women’s) sexuality and SRHR in Pakistan. The general silence when asked about SRHR-related topics, including child marriage, and the need to consistently probe during data collection was indicative of a limited space for youth to express themselves. Nevertheless, the study found a few cases of resistance where youth, particularly *Kirans*, intervened in child marriages. The *Kirans’* ability to do so could be attributed to the empowerment and knowledge-based training they received as part of the Yes I Do programme. These trainings included raising awareness on SRHR issues–including gender equality and child marriage, skill-building on self-expression, engaging with community members, and intervening in cases of child marriage. This presents an opportunity, also relevant to young men, where educated women who delay their marriage become role models in their community [46].

As per our study findings, young people still prefer to marry young, even though the preferred age may be above 18 years. Many of the decision-making dynamics documented around marriage prior to 18 years could also apply to marriage occurring past the 18 year mark. Consent and decision-making are important issues to focus on, irrespective of the strict legal threshold of 18 years, particularly because the age of first marriage is rising in Pakistan. Hence, attention is needed to work with young people who may be above 18 years, but are still in a marriage at a relatively young age. It is critical to ensure that they are empowered to make choices that benefit their aspirations and well-being.

In the Pakistani context, many young people do not have freedom to express themselves. While the topic guides were translated in Urdu and Sindhi languages, pre-tested and adjusted to be culturally appropriate, youth were shy–particularly those with lower education levels. Despite considerable probing, in some cases, young people remained silent when asked about SRHR-related topics, including child marriage. The participants also included more educated young people, and some of them had undergone Yes I Do empowerment-based trainings. These youth were more likely to speak candidly and were more aware about the negative consequences of child marriage. The inclusion of these empowered young people could have influenced the findings, i.e. in other areas, a similar study could have had (slightly) different outcomes. The fathers included in the FGD were relatively well-educated which could have influenced their perspectives. In addition, prior research has found exchange marriages to be prevalent in Sindh, however, it was not prominent in our findings and in other research conducted in the same areas [5]. Lastly, although young people who were married as children were included in the study sample, the study did not dive deeper into their lived experiences after marriage.

## Conclusion

The findings confirm that child marriage is used by families as a protective strategy in a context of economic insecurity. It is also used to protect young women from sexual exploitation and to prevent consensual sexual relations prior to marriage. Young people, particularly women, have limited freedom to make decisions about marriage, due to strict norms that dictate obeying elders and limit young women to childbearing and domestic roles. While parents asking for young people’s consent to marriage may be tokenistic, this could illustrate progress in a context where parent-arranged marriages are the norm. Education is perceived and used, by youth and adults alike, to negotiate decisions within the family and resist community norms in order to delay marriage. Our results suggest that advocating for girls’ education and expanding their livelihood options should be a key and continued focus, while working on social norm change by engaging parents and empowering youth.
